# Breastfeeding During and After Breast Cancer Diagnosis—A Systematic Review of the Literature

**DOI:** 10.3390/jcm14207450

**Published:** 2025-10-21

**Authors:** Anna Ampatzi, Nikoleta Aikaterini Xixi, Rozeta Sokou, Eleni Karapati, Zoi Iliodromiti, Paraskevi Volaki, Styliani Paliatsiou, Nicoletta Iacovidou, Theodora Boutsikou

**Affiliations:** 1 School of Medicine, National and Kapodistrian University of Athens, 11528 Athens, Greece; midwifeanne@med.uoa.gr; 2 Neonatal Department, Aretaieio Hospital, School of Medicine, National and Kapodistrian University of Athens, 11528 Athens, Greece; nerinaxixi@med.uoa.gr (N.A.X.); helenakar@med.uoa.gr (E.K.); ziliodromiti@med.uoa.gr (Z.I.); voulavolaki@med.uoa.gr (P.V.); stpaliatsiou@med.uoa.gr (S.P.); niakobid@med.uoa.gr (N.I.)

**Keywords:** lactation, breast cancer survivor, postpartum period, pregnancy-associated breast cancer

## Abstract

**Background/Objectives:** Breast cancer diagnosis in lactating women is relatively uncommon. The term Pregnancy-Associated Breast Cancer (PABC) refers to breast cancer diagnosed during pregnancy or within the first year postpartum. There are several factors that limit the ability to breastfeed. Despite emerging evidence suggesting that breastfeeding may be feasible and should be supported in women with PABC, there is still limited evidence regarding the percentage of them who attempt breastfeeding, and the challenges they may encounter. This study aims to systematically reviewing the literature on the available evidence regarding breastfeeding in women diagnosed with PABC. **Methods:** PubMed and Scopus were systematically searched for studies on breastfeeding in PABC until 26 June 2025. Data on breastfeeding outcomes and diagnostic challenges in relation to PABC were extracted. The systematic review is registered in PROSPERO (CRD420251043141). **Results:** A total of 15 studies met the inclusion criteria and were included in this review. The results showed a scarcity of literature regarding the percentage of women with PABC who breastfeed. Existing data revealed that a small percentage successfully breastfeed. A common occurrence throughout the studies was the concern of breastfeeding during cancer treatment. In almost all cases, reduced milk production was reported, along with the co-administration of formula as a substitute for breast milk. **Conclusions:** Management of women with PABC should not be limited to oncologic treatment but should also encompass structured breastfeeding counseling and multidisciplinary support, ideally within specialized breast cancer centers. Such integrated care has the potential to optimize maternal health outcomes, improve quality of life, and promote a more favorable pregnancy and postpartum experience.

## 1. Introduction

Pregnancy-associated breast cancer (PABC), defined as breast cancer diagnosed during pregnancy or within one year postpartum, is a rare entity but with increasing prevalence [1]. Despite being rare, PABC is a clinical and diagnostic challenge, as it combines the need for effective treatment with the protection of fetal health and development [2]. With increasing maternal age in pregnancy, particularly in high-income countries, the incidence of PABC is on the rise, and is currently estimated to occur approximately 1 in 3000 pregnancies, while breast cancer is the most common malignancy associated with pregnancy, accounting for up to 21% of all pregnancy-related cancers [3]. The mean age at diagnosis is 33 years, with more than 15% of cases reported in women under the age of 35 [3]. The physiological changes occurring in the breast during pregnancy and lactation, including increased vascularization, lobuloalveolar proliferation, and glandular density, make the diagnosis of PABC a challenge [4]. These structural and hormonal changes can obscure palpable masses and reduce the sensitivity of standard imaging modalities, such as mammography, highlighting the need for careful clinical evaluation and consideration of adjunct imaging such as ultrasound or MRI. These changes may complicate clinical and imaging assessment, often resulting in delays in the diagnosis from one to two months [4]. Consequently, breast cancer is in many cases diagnosed at more advanced stages, which worsens patient prognosis. Furthermore, delays or misdiagnoses are frequently reported among breastfeeding women, as symptoms such as mastitis or galactoceles can mask malignancy, emphasizing the need for heightened clinical suspicion [3,4].

Treatment of PABC poses a unique clinical challenge, as the well-being of the mother and the child needs to be taken into consideration [1]. The therapeutic management of PABC largely follows the general principles of breast cancer treatment in non-pregnant women. However, treatment choices are modified according to the trimester of pregnancy, the stage of the disease, and the tumor characteristics [5]. Surgery remains a fundamental component of therapy and can be performed safely during pregnancy. As to the decision between total mastectomy or breast-conserving surgery, this depends mainly on the disease stage and the timing of diagnosis. Radiotherapy is avoided during pregnancy due to fetal risks and is usually postponed until after delivery. Chemotherapy can be administered relatively safely in the second and third trimesters but is contraindicated in the first trimester because of the increased risk of congenital malformations. Multidisciplinary treatment may also affect lactation, limiting the possibility of breast-feeding either during or after therapy. It is important to note that careful scheduling of chemotherapy cycles around breastfeeding windows and early involvement of lactation consultants can help optimize milk production when possible [2,6].

Breastfeeding management in women with PABC represents an additional area of concern. Breastfeeding may be continued under specific circumstances, particularly when treatment is initiated after delivery; however, weaning is often required due to medications excreted in breast milk. Chemotherapeutic agents, for example, can be toxic to the infant, and a waiting period corresponding to the drug’s half-life is necessary before breastfeeding can be safely resumed. Similarly, targeted therapies and hormonal treatments, such as monoclonal antibodies against HER2 and hormone inhibitors, are contraindicated during pregnancy, and breastfeeding is not recommended during their administration [2].

Furthermore, studies showed that the rate of successful breastfeeding is significantly lower in women who receive chemotherapy either during pregnancy or immediately postpartum, whereas women who were not exposed to systemic therapy had considerably better breastfeeding outcomes [7]. Breast cancer diagnosed postpartum, even within five years after delivery, is increasingly recognized as a more aggressive form of the disease. In these cases, women may be breastfeeding at the time of diagnosis, making management even more complicated. The use of medications such as cabergoline can help in weaning, and women should also be informed about the option of donor human milk [5]. Evidence also indicates that psychological support and structured counseling interventions improve breastfeeding confidence and adherence in this population, addressing maternal anxiety associated with cancer diagnosis and lactation challenges [2].

Breastfeeding plays a dual role in breast cancer. On one hand, long-term breast-feeding is associated with a 7% reduction in breast cancer risk and up to 20% reduction in triple-negative breast cancer incidence [8]. On the other hand, women in the postpartum period are at increased risk for aggressive breast cancer types, including luminal B and triple-negative tumors, especially when diagnosis occurs within five years of childbirth [9]. Lastly, the administration of chemotherapy and targeted therapies during lactation may require the cessation of breastfeeding until termination of treatment [2].

In addition, emerging data suggest that partial breastfeeding, expression and storage of milk, and multidisciplinary care planning can allow some level of lactation while minimizing treatment interruption, although standardized protocols are lacking [2].

This study aims to review the available literature on various aspects of breastfeeding in the context of PABC. Specifically, it explores the obstacles to the initiation and continuation of breastfeeding among women diagnosed with PABC, and the diagnostic challenges in breastfeeding women. It also examines the short-term and long-term effects of breastfeeding on cancer prognosis and treatment outcomes, and quality of life. Finally, the study investigates how different treatment options, such as surgery, chemotherapy, and radiotherapy, affect breastfeeding and lactation outcomes among PABC patients and survivors.

## 2. Materials and Methods

### 2.1. PRISMA Statement

This systematic review was developed in accordance with the PRISMA (Preferred Reporting Items for Systematic Reviews and Meta-Analyses) guidelines [10]. Inclusion criteria, data synthesis methods, and results were predefined in a protocol registered in PROSPERO (CRD420251043141) and are available online [11].

### 2.2. Search Strategy

PubMed and Scopus were systematically researched from 1 April until 6 June 2025, and all relevant articles in English were retrieved with no geographical or time restrictions. Two search phrases, one for each database, were formulated using pre-specified key words and Boolean operators: (breast cancer OR breast cancer survivors OR breast cancer patients OR breast neoplasms) AND (lactating women OR pregnant women OR pregnancy OR lactating period OR postpartum period) AND (breastfeed OR breastfeeding).

### 2.3. Eligibility Criteria

Two independent authors (AA, EK) reviewed the retrieved articles for inclusion, and any discrepancies were discussed and solved. A third reviewer assessed the selected studies for eligibility (NAX). The target population included women during pregnancy or in the postpartum period. Since the review focused on studies exploring the relationship between breastfeeding and PABC, all studies reporting on breastfeeding outcomes in the population of interest were assessed for inclusion in the study. Studies written in either English or Greek were considered eligible. Randomized controlled trials, observational studies, case–control studies, cross-sectional studies, and case studies or case-series reports were assessed for inclusion, regardless of the sample size or setting.

Studies not published in English or Greek, or involving nulliparous women, or focusing exclusively on postmenopausal breast cancer, and those addressing breast cancer occurring not in pregnancy or lactation were excluded from this review, as well as reviews, meta-analyses, comments, and editorials.

### 2.4. Data Extraction

For data extraction, collective tables were compiled, which included data on the first author, publication year, country, study type, study duration, study design, study population, and key findings/outcomes. One author (AA) independently extracted article data, and the complete table was revised by two other authors (EK, NAX).

### 2.5. Risk of Bias Assessment

Two authors (NAX and AA) independently evaluated the risk of bias for the included studies and resolved any discrepancies through discussion. For observational studies, the CLARITY Group’s Tool to Assess Risk of Bias in Cohort Studies from McMaster University, Hamilton, ON, Canada, was employed. This tool comprises eight questions, each with four possible response options. For qualitative studies, we used the Critical Appraisal Skills Programme (CASP) checklist for qualitative research. The tool consists of ten questions designed to guide the appraisal of study quality and rigor. Detailed description of the tool can be found in the Supplementary Materials.

## 3. Results

**Figure 1:**
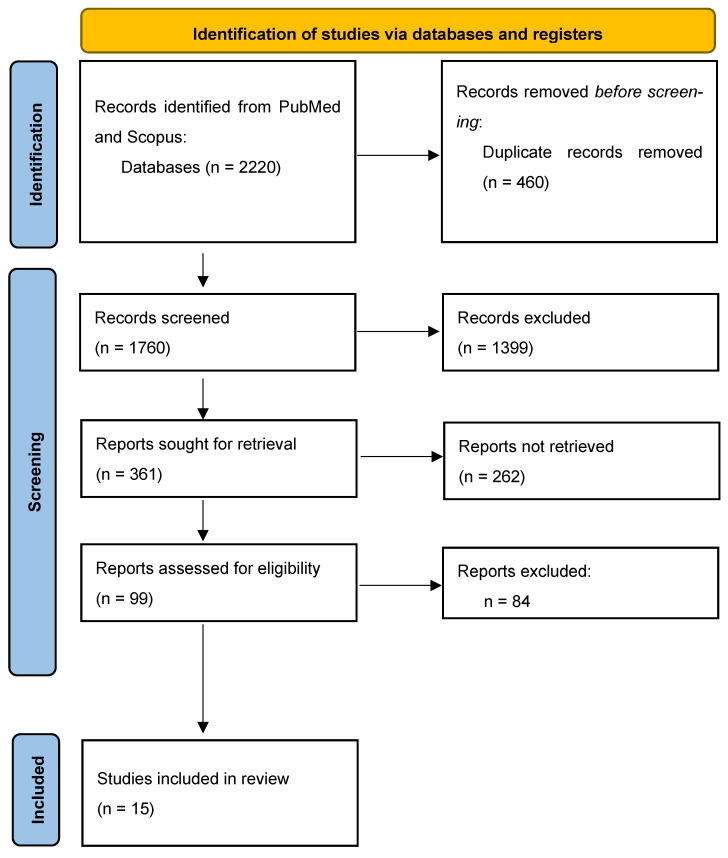
PRISMA flow diagram.

A total of 2220 initial articles were retrieved from PubMed and Scopus. From this total, 15 studies met the eligibility criteria and were included in this systematic review [5,7,12,13,14,15,16,17,18,19,20,21,22,23,24]. Figure 1 shows the flow chart for the inclusion of studies.

The included studies primarily focused on women diagnosed with PABC, defined as breast cancer diagnosed during pregnancy or within 12 months postpartum. Table 1 and Supplementary Tables S1 and S2 summarize the characteristics and the risk of bias assessment of the included studies. Study types included retrospective cohort studies [12,18,19] and prospective cohort studies [5,20,21,22,23]. Qualitative studies, which explored patient experiences, decision-making, and psychosocial impacts, were performed across multiple high-income countries, including the USA [13,17,24], Australia [14], and Italy [7,16].

### 3.1. Diagnosis During Pregnancy and Lactation

Younger women, who are the majority of PABC cases, are less likely to undergo routine screening. U.S. guidelines do not recommend population-based screening before the age of 40 years, unless risk factors such as BRCA mutations, TP53, CHEK2, family history, or prior chest radiation exist [18]. Ultrasound is considered the initial diagnostic modality for pregnant and lactating women presenting with a breast mass. Studies by Jafari et al. [19] and Nissan et al. [21] emphasized the utility and limitations of imaging in this population. Jafari et al. identified high-resolution ultrasound as the first-line method for evaluating symptomatic women and highlighted its safety and high sensitivity. Nissan et al. demonstrated that the visibility of breast tumors on dynamic contrast-enhanced (DCE) MRI is significantly reduced during lactation due to intense background parenchymal enhancement (BPE), though the addition of diffusion tensor imaging (DTI) may improve tumor detection and quantitative characterization. Similarly, Hu et al. [18] reported that most PABC malignancies presented with concerning characteristics (irregular shape) on imaging (82.6%), and that 84.6% of cases detected through prevention programs were identified on mammography or combined mammography and MRI. Lee et al. [5] reported that the median age at diagnosis is 34 years, with stage distribution of I (28%), II (44%), III (23%), and IV (5%), noting that the majority of tumors were high grade (74%), 59% were estrogen receptor positive, and 31% were HER2 positive. On the contrary, Connell et al. [14] observed that advanced PABC was often diagnosed using mammography due to tumor morphology, and Adeniji Sofoluwe et al. [12] reported that abnormal nipple secretions were the most common presenting symptom in pregnant and lactating women. Sullivan et al. [23] reported that PABC was diagnosed at either stage I (27%) or stage II (73%) with a mean age of 31.7 years. Study findings regarding the diagnosis of PABC are demonstrated in Table 2.

### 3.2. Disease Characteristics

PABC tends to present at more advanced stages, compared with non-pregnant women of similar age, and is often aggressive [5,21]. Lee et al. [5] found that 74% of patients had grade 3 tumors, Hu et al. [18] reported that 88% of symptomatic women presented with invasive disease, and Adenji-Sofoluwe et al. [12] confirmed that a large portion of PABC cases present with palpable masses and are node positive at diagnosis. This was mainly attributed to the physiological changes in the breast during pregnancy, which can mask the symptoms and delay the diagnosis [19]. A study noted delayed diagnosis, especially in low-resource settings (e.g., Dusengimana et al.), due to low awareness and limited access to imaging [15]. Regarding tumor characteristics, PABC tends to be characterized by high-grade tumors, hormone receptor negativity (ER-/PR-), and HER2 overexpression, suggesting a poorer prognosis compared to typical breast cancer in young women [5,18]. The results are summarized in Table 3.

### 3.3. PABC Treatment and Breastfeeding

The management of PABC poses unique challenges, requiring careful consideration of maternal and fetal well-being while maintaining optimal oncologic outcomes. Treatment generally follows the standard protocols for breast cancer beyond pregnancy, with necessary modifications in therapy type and timing guided by gestational age to ensure fetal safety and reduce risks to the developing child. According to Adeniji-Sofoluwe [12], the overall aim of breast cancer treatment in pregnant or breastfeeding patients is to achieve local control of the disease and prevent systemic metastases. Neoadjuvant chemotherapy may be particularly suitable since breast cancers associated with pregnancy are often diagnosed at a more locally advanced stage. In such cases, treatment is carefully adjusted to minimize harm to the fetus, and therefore, delaying radiation therapy to the breast and chest until after the completion of pregnancy may be a viable option. Stopenski et al. [22] evaluated breastfeeding outcomes in women whose chemotherapy was either paused or completed at least three weeks before delivery, and reported that among 96 women diagnosed with PABC, all attempted breastfeeding, but of the 74 women who received chemotherapy, only 34.5% exclusively breastfed, while 63.5% faced breastfeeding difficulties. It is worth mentioning that those who started chemotherapy earlier in pregnancy had greater challenges with milk production. Chemotherapy initiated at 17 weeks was associated with a 75% chance of reducing milk production compared to a 50% chance when treatment was started at 24 weeks, while gestational age at the last treatment or at delivery did not significantly affect breastfeeding success. Similarly, Dusengimana et al. [15] reported that 11 out of 12 women with PABC received treatment, though three experienced delays or modifications due to pregnancy or breastfeeding, and four had to discontinue breastfeeding altogether to initiate therapy. Surgical management also showed variation across studies: Gorman et al. [17] reported that 7 out of 11 patients underwent tumor excision surgery, while 4 out of 11 had mastectomies. Higgins et al. reported that all patients in their study underwent breast-preserving surgery. Adjuvant therapies were also common in these populations, and Gorman et al. [17] reported that 9 out of 11 patients received adjuvant chemotherapy, 8 out of 11 received radiotherapy, and 4 out of 11 were given antiestrogen treatment. Higgins et al. [24] reported that 7 out of 11 patients underwent radiotherapy and 3 out of 11 received chemotherapy. Lee et al. [5] further observed that 23 patients (59%) underwent surgery during pregnancy, including 4 (17%) in the first trimester, with the majority (61%) receiving tumor excision surgery, 35% unilateral mastectomy, and 4% bilateral mastectomy; in addition, chemotherapy was more frequently administered in the second or third trimester, accounting for 51% (20 out of 39 patients). Breastfeeding outcomes also varied significantly depending on treatment type and timing. Sullivan et al. [23] reported that only 18 out of 40 women (45%) initiated breastfeeding, and initiation rates were lower (37.9%) among those who received postpartum therapy. Lee et al. [5] reported an even lower initiation rate, only 3 out of 31 women (10%) breastfeeding, and none of those who had received chemotherapy during pregnancy were able to breastfeed. Stopenski et al. [22] similarly revealed that 91% of women who attempted breastfeeding without chemotherapy were successful, compared to only 37% of those who received chemotherapy, most often due to unsuccessful lactation, a finding that was also supported by Azim et al. [7]. On the other hand, Azulay Chertok et al. [13] reported more positive results, with 80% of participants either breastfeeding or expressing milk. Dusengimana et al. [15] reported that 9 out of 12 women with PABC were able to breastfeed, though some were forced to interrupt due to ongoing treatment. In the studies by Gorman et al. and Higgins et al. [17,24], successful breastfeeding was shown to be feasible, particularly from the healthy breast, for women following treatment, although breastfeeding success was notably lower among those who had received radiotherapy or undergone surgery. The results of the included studies on the effect of PABC treatment on breastfeeding are shown in Table 4.

### 3.4. Breastfeeding Outcomes

Breastfeeding post-treatment was rarely successful (Table 5). In the study by Higgins et al. [24], only a few women lactated from the treated breast. While treatment is a main aggravating factor, breast cancer itself can significantly impair a woman’s ability to breastfeed, mainly due to physical changes in the breast tissue and asymmetry between breasts. Gorman et al. [17] reported that many survivors faced insufficient milk supply, and also found breastfeeding as physically and emotionally demanding. Azulay Chertok et al., among others [13,24], reported that many women reported breast asymmetry, which often led to discomfort and breastfeeding difficulties. Azim et al. [7], reported that women who had breast-conserving surgery had reduced milk supply from the other breast and increased nipple discomfort and/or pain.

### 3.5. Psychological Impact and Sociocultural Context

Breastfeeding decisions are made by a complex interplay of psychological, social, and cultural factors. Women with PABC encounter unique emotional and physiological challenges that may influence their willingness and ability to initiate and maintain breastfeeding. Studies, such as Connell et al. [14] and Gorman et al. [17] highlighted maternal distress, feelings of loss, and fear of harming the infant as common emotional responses. Specifically, in the study by Connell et al. [14], Australian women with breast cancer had increased concern about fertility, contraception, pregnancy, and breastfeeding. These issues were often amplified by the lack of consistent information from healthcare professionals, which led to increased maternal distress and feelings of loss and further complicated decisions on breastfeeding and treatment. In low-resource settings, as reported in the study by Adeniji-Sofoluwe et al. [12], limited diagnostic access and cultural norms influenced diagnosis timing and maternal decisions. The researchers also noted that while benign breast diseases, like fibroadenomas, were more common among pregnant and lactating women, the presence of malignant lesions could be misinterpreted as normal lactation changes.

## 4. Discussion

This systematic review includes 15 studies on breastfeeding in women with PABC and underlines the challenges faced by women breastfeeding following a breast cancer diagnosis during pregnancy or the postpartum period. This diagnosis imposes difficulties-medical, psychological, and social- in the breastfeeding efforts of this vulnerable group.

According to the World Health Organization, exclusive breastfeeding is recommended for the first six months of an infant’s life. A small proportion of mothers will be diagnosed with breast cancer either during pregnancy or in the postpartum period. The stage at diagnosis varies, as the breast is highly sensitive and undergoes physiological changes during this life stage [2].

Our study revealed delays or misdiagnoses of breast cancer in young breastfeeding women, by primary care physicians or by specialists. This is particularly concerning given that pregnancy- and lactation-associated breast cancer (PABC) often presents with clinical features that overlap with benign lactation-related changes, such as increased breast size, nodularity, or mastitis-like symptoms, which can mask malignant findings [19,21]. Multiple studies indicate that such delays are common, with median times from first symptom to diagnosis ranging from 24 to 109 days, and in some populations extending up to 212 days from initial healthcare contact to confirmed diagnosis [15,18]. These delays often result from both misinterpretation of symptoms by primary care providers and limited initial imaging, as palpable masses are frequently attributed to benign conditions such as galactoceles, mastitis, or fibroepithelial lesions [12,19]. Diagnostic imaging is further complicated by physiological changes in the lactating breast, including increased vascularity, parenchymal density, and heterogeneous tissue, which reduce the sensitivity of mammography and make ultrasound interpretation more challenging [21,24]. Notably, in several cohorts, a significant proportion of women were initially misdiagnosed or experienced delayed recognition by specialists, highlighting the need for heightened clinical suspicion, timely use of imaging such as breast ultrasound or MRI when indicated, and prompt referral for biopsy of persistent or suspicious lesions [18,23].

Ultrasound is a sensitive diagnostic tool in pregnant and lactating women and is the preferred imaging method due to increased breast density during pregnancy or lactation. The most common ultrasound finding is an irregular mass with posterior acoustic enhancement. Cystic elements may also be observed. In most of the studies included in the present review, similar findings were reported, such as irregular masses with cystic elements. Needle biopsy and/or drainage may be an appropriate initial procedure, with a sensitivity of 90% [4].

If suspicious findings are present or if ultrasound is inconsistent with the clinical examination, further imaging with mammography or digital breast tomosynthesis (DBT, or 3D mammography) may be necessary. Mammography or DBT can detect architectural anomalies and calcifications not visible on ultrasound and can determine the disease extent in malignancy cases. Core needle biopsy, rather than fine-needle aspiration, should be performed after a comprehensive imaging evaluation. Ultrasound-guided biopsy is generally performed for palpable masses. If the mass is not visualized on ultrasound, a stereotactic mammography-guided or MRI-guided biopsy may be recommended. Lactating women should also be informed of the slightly increased risk of bleeding due to hyperemia. Discontinuation of breastfeeding prior to biopsy is not recommended, as abrupt cessation may increase inflammation and fistula formation. If a woman is diagnosed with breast malignancy during initial imaging and biopsy, further biopsy of suspicious regional lymph nodes (axillary, internal mammary, supra-/infraclavicular) may be recommended [25].

Currently, there are no reliable epidemiological data specifically on breastfeeding among women with a history of breast cancer. Guidelines from the Society of Obstetricians and Gynecologists of Canada (SOGC) emphasize that women with a history of breast cancer should be encouraged to breastfeed, as there is no evidence that breastfeeding increases the risk of recurrence or development of a second cancer, nor does it pose risk to the infant [26,27]. Observational data suggest that breastfeeding may have a protective effect against breast cancer in the general population, potentially through hormonal changes that delay the resumption of menstrual cycles, thereby reducing cumulative exposure to estrogen, and through breast cell apoptosis that may eliminate cells with DNA damage [28,29], though these benefits have yet to be specifically quantified in women with PABC.

Medically, while breastfeeding is not universally contraindicated in PABC, the necessity of treatments such as chemotherapy, radiotherapy, or surgical interventions can interfere with breastfeeding success. Breast-conserving surgery or mastectomy involving the lactating breast often compromises milk production [30]. Surgery is considered the treatment of choice and can be performed in any trimester of pregnancy. Radical mastectomy is usually performed, as breast-conserving surgery requires radiotherapy, which is contraindicated during pregnancy. Axillary lymph node dissection is indicated when nodes are clinically positive or infiltrated (confirmed by FNAC). Sentinel lymph node biopsy is an option in node-negative disease. Systemic chemotherapy is avoided during the first trimester due to teratogenic risk, but if indicated, it can be administered during the second and third trimesters, with treatment discontinued by 35 weeks or three weeks before delivery to reduce maternal and fetal risks. Radiotherapy, endocrine therapy, and biologic therapy are only used postpartum due to adverse fetal effects [3]. Certain chemotherapeutic agents, such as anthracyclines and taxanes, can be safely used during the second and third trimesters, whereas tamoxifen and trastuzumab are contraindicated during pregnancy [31]. Imaging for diagnosis should be adapted to minimize fetal exposure, with ultrasound as first-line evaluation and mammography with shielding if necessary. These considerations highlight that the ability to breastfeed is strongly influenced by the timing and type of cancer treatment, and careful prenatal planning, individualized treatment schedules, and close monitoring of both maternal and fetal well-being are essential to optimize breastfeeding outcomes [31,32].

Postpartum breastfeeding management follows the principles used for women diagnosed during pregnancy and may include oncologic surgery, chemotherapy, adjuvant anti-HER2 therapy, or hormone therapy. Women who require treatments that are contraindicated during lactation and choose to stop breastfeeding may benefit from dopamine agonists, such as cabergoline [2,25]. Currently, women are advised not to breastfeed during certain breast cancer treatments, such as chemotherapy or hormone therapy. However, breastfeeding may be achievable from one or both breasts after treatment. “Pump and dump” strategy can maintain milk production until treatment is completed [1].

Apart from medical reasons, the literature also highlights the importance of psychological factors. Women with PABC often experience distress from the perceived loss of the maternal role associated with breastfeeding [17]. They must deal with anxiety and stress related to the health of their infants, fear of disease recurrence, and feelings of guilt. The present study underlines the importance the need of proactive counseling about breastfeeding and lactation in women with PABC. Standardized protocols are still not available in many oncology settings. Healthcare providers could help alleviate maternal anxiety and facilitate informed decision-making [2,33]. Furthermore, post-treatment counseling and support should address not only the physical recovery but also the emotional well-being of the affected mothers [9,33].

Despite these challenges, the review indicates that with appropriate counseling and individualized care plans, breastfeeding may still be achieved in women with PABC. For example, breastfeeding from the unaffected breast is often possible, especially if treatment has not yet begun or if lactation is restored after therapy. Proper timing of chemotherapy and surgery can help create time periods during which breastfeeding is safe. Several studies suggest that prompt implementation of maternal consultation may further lead to successful breastfeeding [31,34]. A systematic review by Bhurosy et al., which aimed at evaluating breastfeeding engagement and at identifying factors that influence breast-feeding among breast cancer survivors, in line with our findings, highlighted the substantial challenges these women face when attempting to initiate breastfeeding. Although breast-conserving surgery and adjuvant radiotherapy may compromise the lactational capacity of the ipsilateral breast, some residual functional tissue often remains capable of milk production. Survivors frequently encounter additional barriers, including uncertainty regarding the safety and feasibility of breastfeeding, inadequate guidance from healthcare providers, limited psychosocial and family support, and restricted access to International Board-Certified Lactation Consultants [35]. Emerging evidence also suggests that individualized lactation planning, early referral to lactation consultants, use of breast pumps to maintain milk production during treatment interruptions, and scheduling treatment cycles around breastfeeding windows can improve outcomes [34,36]. Moreover, patient education regarding partial breastfeeding, milk expression safety during therapy, and psychosocial support can mitigate maternal anxiety and increase confidence, yet standardized protocols for lactation support in oncology settings remain limited, with few studies providing guidance on optimizing milk output while minimizing treatment interference [2,37].

This study has limitations. Firstly, all included studies were designed as observational cohorts, which are prone to residual confounding, but most importantly, the available literature regarding our research question was limited. Secondly, the heterogeneity among included studies regarding study design, sample size, data collection methods, and analysis limited the ability to perform a meta-analysis and made direct comparisons challenging. Also, most of the studies had small sample sizes, which may affect the reliability of the findings. On the other hand, language restrictions may have led to the exclusion of relevant studies. Lastly, although the formal risk-of-bias assessment indicated that while most studies were low or probably low risk across key domains, some studies had higher risk in follow-up and co-intervention domains, which may have contributed to variability in reported breastfeeding outcomes and maternal experiences. These methodological considerations further emphasize that the findings should be interpreted cautiously and highlight the need for individualized care when applying these results to clinical practice. Future research must continue to unveil this under-investigated condition. Longitudinal studies to assess breastfeeding outcomes after PABC treatment, safety evaluations of newer oncology drugs during lactation, and patient-centered trials involving breastfeeding interventions in cancer care settings are all needed.

## 5. Conclusions

This review indicates that breastfeeding is possible for some women with PABC, though often suboptimal. Most women must rely on the untreated breast for milk production, thus making the need for targeted prenatal counseling and postnatal support clear. Survivors face unique breastfeeding challenges and require multidisciplinary guidance. Delays or misdiagnoses are common in this population, as physiological changes during pregnancy and lactation can mask malignant findings, often leading to late detection and more advanced disease at diagnosis. Since PABC is a rare entity, clinical guidelines remain limited, although, when safe, breastfeeding between delivery and treatment initiation can be beneficial. More research is needed to guide care, especially regarding lactation safety during active cancer, and to develop strategies for earlier recognition and timely management of PABC.

## Figures and Tables

**Table 1:** Study characteristics.

First Author	Year	Country	Study Type	Study Period	Population, N
Adeniji-Sofoluwe	2015	Nigeria	Retrospective study	2006–2013	21
Azim	2010	Italy	Qualitative study	1990–2005	32
Azulay Chertok	2020	USA	Qualitative study	2017–2019	20
Connell	2006	Australia	Qualitative study	2000–2002	13
Dusengimana	2018	Ruanda	Retrospective cohort study	2012–2014	12
Faccio	2020	Italy	Qualitative study	2016	38
Gorman	2009	USA	Qualitative study	1995–2000	11
Higgins	1994	USA	Qualitative study	1965–1989	13
Hu	2020	USA	Retrospective cohort study	2019	145
Jafari	2023	Iran	Retrospective cohort study	2015–2021	75
Lee	2019	USA	Prospective cohort study	2006–2016	39
Lööf-Johanson	2011	Sweden	Prospective cohort study	1988–1992	250
Nissan	2020	Israel	Prospective cohort study	2016–2018	11
Stopenski	2017	USA	Prospective cohort study	1998–2013	96
Sullivan	2022	Australia	Prospective cohort study	2013–2014	40

**Table 2:** Diagnosis during pregnancy and lactation.

First Author	Results
Adeniji-Sofoluwe	Abnormal nipple secretions were the most common manifestation during pregnancy and lactation
Connell	The diagnosis was made using mammography at advanced cancer stages due to its morphology
Dusengimana	There were no significant differences in delay of diagnosis or stage in diagnosis among women with PABC and non-PABC
Higgins	Diagnosis during early stages
Hu	Out of the 9.1% of the patients diagnosed with PACB through prevention programs, 84.6% was diagnosed with mammography or mammography combined with MRI. The majority of the malignancies appeared with concerning characteristics on imaging (82.6%)
Jafari	First line diagnostic modality for evaluation of the breast, especially in symptomatic women is high resolution ultrasonography
Lee	The median age of diagnosis was 34 years. The Stage distribution was the following: I, 28%; II, 44%; III, 23%; και IV, 5%. 74% of patients (29/39) had grade 3 tumors, 59% (23/39) was Estrogen Receptor positive, and 31% (12/39) was HER2 positive.
Lööf-Johanson	Probable correlations between total breastfeeding duration, parity and age at first pregnancy
Nissan	The visibility of breast cancer on dynamic contrast enhanced (DCE)-MRI is significantly reduced during lactation due to intense background parenchymal enhancement (BPE). However, the additional application of Diffusion Tensor Imaging (DTI) may improve both the imaging and quantitative characterization of pregnancy-associated breast cancer (PABC).
Sullivan	Participants were diagnosed with either stage I (27%) or stage II (73%) PABC at a mean age of 31.7 years

**Table 3:** PABC characteristics.

First Author	Results
Connell	The diagnosis was made by mammography in advanced-stage breast cancer due to the tumor’s morphology.
Dusengimana	There were no significant differences in diagnostic delay or cancer stage at diagnosis between women with pregnancy-associated breast cancer (PABC) and women without PABC.
Higgins	Diagnosis at early stages of breast cancer in pregnant women after they had received their treatment.
Hu	Most breast cancers had high risk characteristics during diagnostic imaging (82.6%)
Lee	Stage I, 28%; II, 44%; III, 23%; και IV, 5%. 74% of patients (29/39) had grade 3 tumors, 59% (23/39) had positive ER, and 31% (12/39) had positive HER2
Sullivan	Stage I (27%) and Stage II (73%)

**Table 4:** PABC treatment and breastfeeding.

First Author	Results
Adeniji-Sofoluwe	Adjuvant and neo-adjuvant chemotherapy
Dusengimana	11/12 women with PABC received treatment, 3 had recorded delays or modifications in the treatment regimens due to the pregnancy or breastfeeding. 4 stopped breastfeeding for treatment initiation
Gorman	Surgery modalities: 7/11 had tumor excision surgery, 4/11 had mastectomies. Therapy modalities: 9/11 had adjuvant chemotherapy, 8/11 had radiotherapy, 4/11 received antiestrogen treatment
Higgins	All patients had breast-preserving surgery. 7/11 patients had also radiotherapy, 3/11 patients received 6 rounds of chemotherapy
Lee	23 (59%) were submitted to surgery during pregnancy, 4 (17%) in the 1st trimester. 61% (14/23) had tumor excision surgery, 35% (8/23) had unilateral mastectomy, and 4% (1/23) bilateral mastectomy. All patients treated with chemotherapy (51%, 20/39) were in the second or third trimester
Stopenski	74 women received chemotherapy during pregnancy, which was completed up to 3 weeks prior to delivery
Sullivan	Intensive treatment during pregnancy: 18/40 Surgical treatment during pregnancy: 23/40 Intensive treatment postpartum: 29/40

**Table 5:** Breastfeeding outcomes.

First Author	Results
Azim	10/32 started breastfeeding, 4/32 ceased withing one month, 6/32 breastfed for more than 6 months. The main causes of breastfeeding failure were uncertainty regarding treatment safety and the contraindication expressed by the oncologist or the gynecologist.
Azulay Chertok	Breastfeeding/pumping after PABC diagnosis: 16 (80%). Mothers expressed fears regarding the nursing of their offsprings.
Connell	All participants had viable pregnancies and expressed intention to breastfeed
Dusengimana	9/12 women breastfed at diagnosis. 3/12 had to delay or modify their treatment due to pregnancy or breastfeeding. 4/12 had to stop breastfeeding to start treatment.
Faccio	All women with PABC experience, regarded breastfeeding as fundamental. Their fear of not being able to breastfeed caused distress.
Gorman	10/11 participants started breastfeeding. All participants had high drive to breastfeed but faced serious challenges.
Higgins	4/13 managed to breastfeed successfully from the healthy breast. In 3/12 lactation was unsuccessful. The following situations were reported: reserved hope, exhaustion from relying only on one breast, motive regardless of challenges, support and lack thereof, external encouragement.
Lee	3/39 women reported successful breastfeeding
Stopenski	45/96 achieved successful lactation and breastfeeding. Women receiving chemotherapy during pregnancy are less likely to breastfeed.
Sullivan	18/23 women breastfed (including those who had undergone surgery)

## Data Availability

Data are contained within the article.

## Supplementary Materials

The following supporting information can be downloaded at: https://www.mdpi.com/article/10.3390/jcm14207450/s1, Table S1: Risk of Bias assessment for observational studies; Table S2: Risk of Bias assessment for qualitative studies; PRISMA checklist; PROSPERO registered protocol.
