# Supplementary material for: Breastfeeding During and After Breast Cancer Diagnosis—A Systematic Review of the Literature
Source: J Clin Med. 2025 Oct 21;14(20):7450. doi: 10.3390/jcm14207450 (PMC12565407; doi:10.3390/jcm14207450)
Supplement: Supplementary file 1 [file jcm-14-07450-s001.zip › PROSPERO.pdf]

NIHR | National Institute for Health and Care Research

PROSPERO
International prospective register of systematic reviews

"Breastfeeding during and after breast cancer diagnosis – A systematic review of literature

NIKOLETA AIKATERINI XIXI, Anna Ampatzi, Helena Karapati

Citation
NIKOLETA AIKATERINI XIXI, Anna Ampatzi, Helena Karapati. "Breastfeeding during and after breast cancer diagnosis – A systematic review of literature. PROSPERO 2025 CRD420251043141. Available from https://www.crd.york.ac.uk/PROSPERO/view/CRD420251043141.

REVIEW TITLE AND BASIC DETAILS

Review title
"Breastfeeding during and after breast cancer diagnosis – A systematic review of literature

Condition or domain being studied
Breast Cancer; Breastfeeding

Rationale for the review
This study aims to systematically review the literature on the available evidence regarding breastfeeding in women diagnosed with breast cancer either during pregnancy or in the postpartum period.Given the rarity of PABC, clinical guidelines remain limited although, when safe, breastfeeding between childbirth and treatment initiation can be beneficial. More research is needed to guide care, especially regarding lactation safety during active cancer.

Review objectives
how does pregnancy assossiated breast cancer affect breastfeeding and lactation?

Keywords
Lactation; Breast cancer survivors; Postpartum period; Pregnancy-associated breast cancer

Country
Greece

ELIGIBILITY CRITERIA

Population

Included
pregnant women with Pregnancy associated breast cancer

Excluded
no pregnancy or no breastfeeding or no cancer

Intervention(s) or exposure(s)

Included
Breastfeeding-lactation

Comparator(s) or control(s)

This review does not have any comparators

Study design

Both randomized and nonrandomized study types will be included.

Included
Randomized controlled trials, observational studies, case-control studies, cross-sectional studies, and case or case-series reports, were assessed for inclusion, regardless of the sample size or setting.

Context

The review focused on studies exploring the relationship between breastfeeding and breast cancer. The target population included women, particularly those who were pregnant or in the postpartum period, with no geographical or time restrictions.

TIMELINE OF THE REVIEW

Date of first submission to PROSPERO

29 April 2025

Review timeline

Start date: 1 April 2024. End date: 30 April 2025.

Date of registration in PROSPERO

04 May 2025

AVAILABILITY OF FULL PROTOCOL

Availability of full protocol

A full protocol has not been written.

SEARCHING AND SCREENING

Search for unpublished studies
Only published studies will be sought.

Main bibliographic databases that will be searched
The main databases to be searched are *PubMed* and *Scopus*.

Search language restrictions
The review will only include studies published in Greek and English.

Search date restrictions
There are no search date restrictions.

Other methods of identifying studies
No other methods will be used.

Link to search strategy
A full search strategy is not available.

Selection process
Studies will be screened independently by at least two people (or person/machine combination) with a process to resolve differences.

Other relevant information about searching and screening
None

DATA COLLECTION PROCESS

Data extraction from published articles and reports
Data will be extracted by one person (or machine) only.

Authors will not be contacted for further information.

Study risk of bias or quality assessment
Risk of bias will be assessed using:

CLARITY and CASP

Data will be assessed by one person (or a machine) and checked by at least one other person (or machine).

Additional information will **not** be sought from study investigators if required information is unclear or unavailable in the study publications/reports.

Reporting bias assessment
Risk of bias due to missing results will not be assessed

Certainty assessment

Certainty of findings will not be assessed

OUTCOMES TO BE ANALYSED

Main outcomes

Diagnosis during Pregnancy and Lactation

Disease Characteristics

Breastfeeding Outcomes

Impact of Treatment on Breastfeeding

Psychological and Emotional Impacts

Sociocultural Context

Additional outcomes

There are no additional outcomes.

PLANNED DATA SYNTHESIS

Strategy for data synthesis

In this study data regarding our research question will be extracted from the original studies into a table and they will be combined in a systematic review of the existing literature.

CURRENT REVIEW STAGE

Stage of the review at this submission

Review stage	Started	Completed
Pilot work	✓	
Formal searching/study identification	✓	
Screening search results against inclusion criteria	✓	
Data extraction or receipt of IPD		

Risk of bias/quality assessment

Data synthesis

Review status

The review is currently planned or ongoing.

Publication of review results

Results of the review will be published in English.

REVIEW AFFILIATION, FUNDING AND PEER REVIEW

Review team members

Dr NIKOLETA AIKATERINI XIXI (review guarantor and contact) National and Kapodistrian University of Athens. Greece.

No conflict of interest declared.

Ms Anna Ampatzi. National and Kapodistrian University of Athens. Greece.

No conflict of interest declared.

Dr Helena Karapati. National and Kapodistrian University of Athens. Greece.

No conflict of interest declared.

Named contact

Dr NIKOLETA AIKATERINI XIXI (nerinajr@gmail.com). National and Kapodistrian University of Athens. Greece.

Review affiliation

National and Kapodistrian University of Athens, School of Medicine

Funding source

Review has no funding and no agreed support from an academic institution and is done in authors' own time.

Peer review

There has been no peer review of this planned review.

ADDITIONAL INFORMATION

Review conflict of interest

Declared individual interests are recorded under team member details.. No additional interests are recorded for this review.

Medical Subject Headings

Female; Lactation; Neoplasms; Pregnancy

SIMILAR REVIEWS

Check for similar records already in PROSPERO

PROSPERO identified a number of existing PROSPERO records that were similar to this one (last check made on 30 April 2025). These are shown below along with the reasons given by that the review team for the reviews being different and/or proceeding.

- Effects of Breastfeeding During Pregnancy on Maternal and Newborn Health and Breast Milk Characteristics: Meta-analysis [published 23 September 2024] [CRD42024589464]. The review was judged **not to be similar**
- Influence of Breastfeeding on Breast Cancer Prevention [published 6 August 2020] [CRD42020155092]. The review was judged **not to be similar**
- Breastfeeding and Risk of Breast Cancer. [published 17 April 2024] [CRD42024532351]. The review was judged **not to be similar**
- Breast cancer and breastfeeding: an updated meta-analysis based on 126 observational studies [published 8 November 2020] [CRD42020213442]. The review was judged **not to be similar**
- Differences in Breast Cancer Risk between White and Black Women in Relation to Breastfeeding Practices in the United Kingdom: A Systematic Review [published 21 July 2023] [CRD42023445059]. The review was judged **not to be similar**
- Prevalence of breastfeeding in disability situations: Systematic review and meta-analysis [published 24 February 2025] [CRD420250654600]. The review was judged **not to be similar**
- Pre-pregnancy body mass index and/or weight gain during pregnancy and breastfeeding success in breastfeeding: systematic literature review [published 16 June 2016] [CRD42016041404]. The review was judged **not to be similar**
- Unlocking the Role of BDNF: A Comprehensive Review on Breastfeeding's Impact [published 14 October 2023] [CRD42023469233]. The review was judged **not to be similar**
- The effect of feeding with symbiotic products and mode of delivery on the development of breast milk and infant gut microbiota during pregnancy and lactation: a systematic review [published 25 December 2023] [CRD42023382606]. The review was judged **not to be similar**
- The effect of breastfeeding on the risk of different breast cancer subtypes in women: a systematic review protocol [published 29 October 2020] [CRD42020210771]. The review was judged **not to be similar**
- Factors influencing breastfeeding of women with pre-pregnancy overweight and obesity: a systematic review [published 5 June 2022] [CRD42022334640]. The review was judged **not to be similar**
- How does the currently available breastfeeding information for father impact breastfeeding continuation and breastfeeding choices? [published 20 December 2021]

[CRD42021292284]. The review was judged **not to be similar**

- THE EFFECT OF BREASTFEEDING INTERVENTIONS DURING PREGNANCY ON BREASTFEEDING SELF-EFFICIENCY: A META-ANALYSIS STUDY [published 31 October 2023] [CRD42023474262]. The review was judged **not to be similar**
- Does exist any associations between breastfeeding in infancy and breast neoplasms in the adulthood [published 7 May 2024] [CRD42024540415]. The review was judged **not to be similar**
- The effects of breastfeeding on the risk of endometriosis in mother and child: a systematic review & meta-analysis [published 12 May 2022] [CRD42022320800]. The review was judged **not to be similar**
- The effectiveness of Real-time Tele-lactation Intervention on breastfeeding outcomes among employed mothers: A Systematic Review and Meta-analysis Protocols [published 5 June 2023] [CRD42023429900]. The review was judged **not to be similar**
- The relationship between reproductive factors and breast cancer subtypes in the United States [published 11 February 2019] [CRD42019122515]. The review was judged **not to be similar**
- Breastfeeding among breast cancer survivors worldwide: a systematic review [published 10 July 2020] [CRD42020163896]. The review was judged **not to be similar**
- Behavioral intervention and the outcomes on breastfeeding practices among working mothers. A systematic review. [published 8 November 2023] [CRD42023469667]. The review was judged **not to be similar**
- The effectiveness of real-time Tele-lactation intervention on breastfeeding outcomes among employed mothers: A systematic review and meta-analysis protocol [published 3 May 2023] [CRD42023420556]. The review was judged **not to be similar**

PROSPERO version history

- Version 1.0, published 04 May 2025

Disclaimer

The content of this record displays the information provided by the review team. PROSPERO does not peer review registration records or endorse their content.

PROSPERO accepts and posts the information provided in good faith; responsibility for record content rests with the review team. The guarantor for this record has affirmed that the information provided is truthful and that they understand that deliberate provision of inaccurate information may be construed as scientific misconduct.

PROSPERO does not accept any liability for the content provided in this record or for its use. Readers use the information provided in this record at their own risk.

Any enquiries about the record should be referred to the named review contact
