# Supplementary material for: Breastfeeding During and After Breast Cancer Diagnosis—A Systematic Review of the Literature
Source: J Clin Med. 2025 Oct 21;14(20):7450. doi: 10.3390/jcm14207450 (PMC12565407; doi:10.3390/jcm14207450)
Supplement: Supplementary file 1 [file jcm-14-07450-s001.zip › Risk of Bias Assessment.pdf]

Data Supplement

Title: Breastfeeding during and after breast cancer diagnosis – A systematic review of literature

Authors: Anna Ampatzi, Nikoleta Aikaterini Xixi, Rozeta Sokou, Eleni Karapati, Zoi Iliodromiti, Paraskevi Volaki, Styliani Paliatsiou, Nicoletta Iacovidou, Theodora Boutsikou

A. METHODS

Risk of bias assessment

For observational studies, we used the Tool to Assess Risk of Bias in Cohort Studies, developed by the CLARITY Group at McMaster University [1]. The tool uses eight questions. The examples beneath the questions are intended to clarify the rationale behind answers in each question.

In each question, four answers were possible:

1.Definitely yes (low risk of bias)

2. Probably yes

3. Probably no

4. Definitely no (high risk of bias)

Q1 Was selection patient and control cohorts drawn from the same population?

Definitely yes: patients were drawn from the same administrative database of patients presenting at the same point of care, over the same time frame (<=3 months).

Probably yes: patients were drawn from the same administrative database of patients presenting at the same point of care, over a similar time frame (>3 but <12 months).

Probably no: patients presenting to different points of care (e.g., multicenter study) or at the same point of care, over a different time frame (>= 12 months)

Definitely no: patients presenting to unspecified points of care.

Q2. Can we be confident in the presence of PABC patients?

Based on the inclusion criteria, a definitely yes was pre-specified as the appropriate answer.

Q3. Can we be confident that the outcome of interest was not present at start of study?

Since the presence of our main outcome of interest (breastfeeding) was a mandatory inclusion criterium, a definitely yes was pre-specified as the appropriate answer.

Q4. Did the study match exposed and unexposed for all variables that are associated with the outcome of interest or did the statistical analysis adjust for these prognostic variables?

Definitely yes: Matching or adjustment for all the prognostic variables.

Probably yes: Matching or adjustment for some prognostic variables.

Probably no: Matching or adjustment for one prognostic variable.

Definitely no: No matching or adjustments for prognostic variables.

Q5. Can we be confident in the assessment of the presence or absence of prognostic factors?

Definitely yes: Data collection on prognostic variables through electronic medical records.

Probably yes: Data collection through database or review of charts.

Probably no: Data collection without demonstration of reproducibility.

Definitely no: Data collection process not stated or no data on prognostic factors regarding intubation/late or no intubation cohorts.

Q6. Can we be confident in the assessment of outcome?

Definitely yes: Outcome assessment performed through independent blind evaluation, secure record linkage, or reference to medical records when sufficient for confirmation.

Probably yes: Outcome assessment performed through medical record review or independent evaluation without blinding, but still reasonably reliable.

Probably no: Outcome assessment based primarily on self-report, or use of medical records without sufficient detail for outcomes requiring more robust confirmation.

Definitely no: Outcome assessment not described, or methods are inadequate to ensure validity of outcome ascertainment.

Q7.Was the follow up of cohorts adequate?

Definitely yes: Median follow up of at least 28 days, or all patients discharged or dead.

Probably yes: Median follow up between 14-28 days, or the majority of patients discharged or dead.

Probably no: Median follow-up between 7 and up to but not including 14 days.

Definitely no: Median follow-up less than 7 days or not stated.

Q8. Were co-Interventions similar between groups?

Due to the nature of the variability concerning the co-interventions between early and late groups (eg. differences in sedation), a probably no was prespecified as the appropriate answer.

For the assessment of the randomised controlled trials, Risk of Bias 2 (RoB2) assessment form was used. The tool uses five domains with up to seven questions each. In each question, five answers were possible:

1.Yes (low risk of bias)

2. Probably yes

3. Probably no

4. No

5. Not identified

After summarising all the results from each of the 5 domains, an overall risk was calculated, with three possible ratings:

1. High

2. Some concerns

3. Low

Supplementary Table S1: Risk of Bias assessment for observational studies

Study	Q1	Q2	Q3	Q4	Q5	Q6	Q7	Q8
Hu	Low	Low	Low	Probably Low	Low	Low	Probably Low	Probably Low
Jafari	Low	Low	Low	Probably Low	Probably Low	Low	Probably High	Probably Low
Lööf-Johanson	Probably Low	Low	Low	Probably Low	Probably Low	Probably Low	Probably Low	Probably Low
Nissan	Low	Low	Low	Low	Low	Low	Low	Probably Low

Stopenski	Probably Low	Low	Low	Probably Low	Probably Low	Probably Low	Probably High	Probably Low
Sullivan	Low	Low	Low	Low	Low	Low	Low	Probably Low

Q, Question; Low = Definitely Yes; Probably Low = Probably Yes; Probably High = Probably No; High = Definitely No

For qualitative studies, we used the Critical Appraisal Skills Program (CASP) checklist for qualitative research []. The tool consists of ten questions designed to guide the appraisal of study quality and rigor. Each question addresses a key aspect of qualitative research, including clarity of the research aims, appropriateness of the methodology, recruitment strategy, data collection, reflexivity, ethical considerations, data analysis, and the overall value of the research. Supplementary Table 2: Risk of Bias assessment for qualitative studies.

In each question there were 3 possible answers

Yes

No

Not Applicable (NA)

Section A: Are the results valid?

Q1. *Clear statement of aims – Was there a clear statement of the research aims?*

Consider: Research goals, importance, and relevance.

Q2. *Qualitative methodology – Is a qualitative methodology appropriate?*

Consider: Whether the study aims to interpret participants' actions or experiences, and if qualitative research suits the research goal.

Q3. *Research design – Was the design appropriate for the research aims?*

Consider: Justification of the research design and method choice.

Q4. *Recruitment strategy – Was participant recruitment appropriate?*

Consider: Selection process, rationale for participant choice, and any discussion on non-participation.

Q5. *Data collection – Was data collected in a way that addressed the research issue?*

Consider: Setting justification, collection methods, modifications during study, data form, and data saturation.

Q6. *Researcher-participant relationship – Was this adequately considered?*

Consider: Researcher reflexivity, potential bias, and how they responded to events during the study.

Section B: What are the results?

Q7. *Ethical issues – Were ethical considerations addressed?*

Consider: Informed consent, confidentiality, participant impact, and ethics committee approval.

Q8. *Data analysis – Was the analysis rigorous?*

Consider: Description of analysis process, theme derivation, data selection, contradictory data, and researcher reflexivity.

Q9. *Clear findings – Is there a clear statement of findings?*

Consider: Explicit findings, discussion of supporting and opposing evidence, credibility checks, and relation to research question.

Section C: Will the results help locally?

Q10. *Value of the research – How valuable is the research?*

Consider: Contribution to knowledge, relevance to practice or policy, identification of research gaps, and transferability of findings.

Supplementary Table S2: Risk of Bias assessment for qualitative studies

Study	Q1	Q2	Q3	Q4	Q5	Q6	Q7	Q8	Q9	Q10	Overall Risk of Bias
Azulay Chertok	Yes	Yes	Yes	NA	Yes	NA	NA	NA	Yes	Yes	Moderate
Connell	Yes	Yes	Yes	NA	Yes	NA	NA	NA	Yes	Yes	Moderate
Faccio	Yes	Yes	Yes	Yes	Yes	NA	NA	Yes	Yes	Yes	Low-Moderate
Gorman	Yes	Yes	Yes	Yes	Yes	Partial	Yes	Yes	Yes	Yes	Low

Higgins	Yes	No	No	Yes	Yes	No	NA	No	Yes	Partial	Moderate-High
Azim	Yes	No	No	Yes	Yes	NA	NA	No	Yes	Yes	Moderate-High

Q, Question; NA, Not Applicable

References

1. CLARITY Group at McMaster University. Tool to Assess Risk of Bias in Case Control Studies. Available online: https://www.clarityresearch.ca/assess-risk-of-bias-in-case-control-studies

2. Lee, G.E.; Rosenberg, S.M.; Mayer, E.L.; Borges, V.; Meyer, M.E.; Schapira, L.; Come, S.E.; Partridge, A.H. Contemporary Management of Breast Cancer during Pregnancy and Subsequent Lactation in a Multicenter Cohort of Young Women with Breast Cancer. Breast J 2019, 25, 1104–1110, doi:10.1111/tbj.13431.

3. Dusengimana, J.M.V.; Hategekimana, V.; Borg, R.; Hedt-Gauthier, B.; Gupta, N.; Troyan, S.; Shulman, L.N.; Nzayisenga, I.; Fadelu, T.; Mpunga, T.; et al. Pregnancy-Associated Breast Cancer in Rural Rwanda: The Experience of the Butaro Cancer Center of Excellence. BMC Cancer 2018, 18, 634, doi:10.1186/s12885-018-4535-y.

4. Hu, X.; Myers, K.S.; Oluyemi, E.T.; Philip, M.; Azizi, A.; Ambinder, E.B. Presentation and Characteristics of Breast Cancer in Young Women under Age 40. Breast Cancer Res Treat 2021, 186, 209–217, doi:10.1007/s10549-020-06000-x.

5. Jafari, M.; Abbasvandi, F.; Nazeri, E.; Olfatbakhsh, A.; Kaviani, A.; Esmaeili, R. Ultrasound Features of Pregnancy-Associated Breast Cancer: A Retrospective Observational Analysis. Cancer Med 2023, 12, 1189 1194, doi:10.1002/cam4.4974.

6. Lööf-Johanson, M.; Brudin, L.; Sundquist, M.; Thorstenson, S.; Rudebeck, C.E. Breastfeeding and Prognostic Markers in Breast Cancer. Breast 2011, 20, 170–175, doi:10.1016/j.breast.2010.08.007.

7. Nissan, N.; Bauer, E.; Moss Massasa, E.E.; Sklair-Levy, M. Breast MRI during Pregnancy and Lactation: Clinical Challenges and Technical Advances. Insights Imaging 2022, 13, 71, doi:10.1186/s13244-022-01214-7.

8. Stopenski, S.; Aslam, A.; Zhang, X.; Cardonick, E. After Chemotherapy Treatment for Maternal Cancer During Pregnancy, Is Breastfeeding Possible? Breastfeeding Medicine 2017, 12, 91–97, doi:10.1089/bfm.2016.0166.

9. Sullivan, E.; Safi, N.; Li, Z.; Remond, M.; Chen, T.Y.T.; Javid, N.; Dickinson, J.E.; Ives, A.; Hammarberg, K.; Anazodo, A.; et al. Perinatal Outcomes of Women with Gestational Breast Cancer in Australia and New Zealand: A Prospective Population-Based Study. Birth 2022, 49, 763–773, doi:10.1111/birt.12642.

10. Adeniji-Sofoluwe, A.T.; Obajimi, G.O.; Obajimi, M.O. Pregnancy Related Breast Diseases in a Developing African Country: Initial Sonographic Evaluation. Pan Afr Med J 2015, 20, 239, doi:10.11604/pamj.2015.20.239.4830.

11.

12. Azulay Chertok, I.R.; Wolf, J.H.; Beigelman, S.; Warner, E. Infant Feeding among Women with a History of Breast Cancer. J Cancer Surviv 2020, 14, 356–362, doi:10.1007/s11764-019-00852-z.
13. Connell, S.; Patterson, C.; Newman, B. A Qualitative Analysis of Reproductive Issues Raised by Young Australian Women with Breast Cancer. Health Care Women Int 2006, 27, 94–110, doi:10.1080/07399330500377580.
14. Faccio, F.; Mascheroni, E.; Ionio, C.; Pravettoni, G.; Alessandro Peccatori, F.; Pisoni, C.; Cassani, C.; Zambelli, S.; Zilioli, A.; Nastasi, G.; et al. Motherhood during or after Breast Cancer Diagnosis: A Qualitative Study. Eur J Cancer Care (Engl) 2020, 29, e13214, doi:10.1111/ecc.13214.
15. Gorman, J.R.; Usita, P.M.; Madlensky, L.; Pierce, J.P. A Qualitative Investigation of Breast Cancer Survivors' Experiences with Breastfeeding. J Cancer Surviv 2009, 3, 181–191, doi:10.1007/s11764-009-0089-y.
16. Higgins, S.; Haffty, B.G. Pregnancy and Lactation after Breast-Conserving Therapy for Early Stage Breast Cancer. Cancer 1994, 73, 2175–2180, doi:10.1002/1097-0142(19940415)73:8<2175::aid-cncr2820730823>3.0.co;2-#.
17. Azim, H.A.; Bellettini, G.; Liptrott, S.J.; Armeni, M.E.; Dell'Acqua, V.; Torti, F.; Di Nubila, B.; Galimberti, V.; Peccatori, F. Breastfeeding in Breast Cancer Survivors: Pattern, Behaviour and Effect on Breast Cancer Outcome. Breast 2010, 19, 527–531, doi:10.1016/j.breast.2010.05.018.
